# Visual Functioning and Mental Health in the Digital Age

**DOI:** 10.3390/jcm14051557

**Published:** 2025-02-26

**Authors:** Vanja Kopilaš, Dora Korać, Lovorka Brajković, Mirko Kopilaš

**Affiliations:** 1 University of Zagreb Faculty of Croatian Studies, 10000 Zagreb, Croatia; dkorac@fhs.hr (D.K.); lbrajkov1@fhs.hr (L.B.); 2 Private Ophthalmology Clinic, 20000 Dubrovnik, Croatia

**Keywords:** digital eye strain, dry eye disease, screen use, mental health

## Abstract

**Background/Objectives**: Considering the omnipresence of digital devices in every aspect of our lives, and from an increasingly younger age, digital eye strain (DES) and dry eye disease (DED) have become a global concern. The main objective of this paper is to conduct a systematic review of the literature on the relationship between digital screen use and ocular surface health, as well as the potential negative impact of impaired visual functioning on certain aspects of mental health and daily performance. **Methods**: Using the PRISMA method, the screening and study selection process resulted in the inclusion of 15 papers published in the electronic databases PubMed and WoS. **Results**: The findings highlight the prolonged use of digital devices and the concerning prevalence of DED or DES symptoms across different populations. A significant relationship was found between DED and DES symptoms and mental health outcomes, including depression, anxiety, and stress. Furthermore, higher frequency and severity of DED or DES symptoms was associated with reduced sleep quality and difficulties in certain aspects of daily functioning. **Conclusions**: Given the ongoing exposure to digital devices and increasing prevalence of DED and DES symptoms across all age groups, there is a need to design interventions and programs focused on preserving ocular surface health and improving subjective well-being. The multifaceted impact these symptoms have on physical and mental health, as well as daily functioning, requires a holistic approach, integrating ergonomic interventions, digital hygiene, and mental health support.

## 1. Introduction

Technological advances over the last two decades have significantly increased access to digital devices such as computers, tablets and smartphones, and have changed our professional and personal routines [1]. It has altered the nature and execution of many work and educational tasks by automating repetitive tasks, improving communication through digital tools, providing unlimited access to information and even changing the way we spend our leisure time [2]. The usage of digital devices in our daily lives became more pronounced during the COVID-19 pandemic. Changes in the educational and work environment, as well as our social functioning due to restrictive measures during the COVID-19 pandemic, have led to even greater use of digital devices [3,4,5,6,7]. Although digital technology has increased our efficiency, provided convenience, and enabled communication with loved ones during the pandemic, this comes at a cost. The use of digital devices for extended periods of time has been linked to numerous ocular, vision-related, musculoskeletal and neurological symptoms [8,9,10,11]. Previous studies have identified prolonged use of digital devices as a significant risk factor for the onset or exacerbation of existing ocular surface disorders, particularly digital eye strain and dry eye disease [7,10,12,13,14].

Digital eye strain (DES), also known as computer vision syndrome (CVS), is usually defined as “the development or exacerbation of recurrent ocular symptoms and/or signs related specifically to digital device screen viewing” [1]. Although prevalence varies by culture, assessment methods and populations observed, some studies have found rates up to 90% [15,16,17]. The symptomatology of DES is heterogeneous and includes symptoms related to the surface of the eye, as well as extraocular symptoms such as neck, shoulder, and back pain [1,18,19]. The most common and disruptive symptoms of DES are redness and dryness of the eyes, headache, itching, burning, blurred vision, and eye pain [2,4,20,21]. Another ocular surface disease caused by prolonged screen exposure is dry eye disease (DED). It is considered one of the most common causes of ophthalmologic consultations, with a prevalence between 11.6% and 50% [22,23]. A systematic review indicated that the prevalence of symptoms related to dry eye disease can be as high as 77.5% [24]. DED is a multifactorial condition characterized by instability in the tear film, which leads to symptoms such as a foreign body sensation, eyelid fatigue, blurry vision, burning, and visual disturbances [25,26]. While there is noticeable overlap in the symptoms of DES and DED, it is essential to differentiate between the two, specifically in terms of duration, causes and symptom presentation. DES is regarded as a temporary condition primarily triggered by prolonged screen exposure, involving both ocular and extraocular symptoms [27]. In contrast, DED is a chronic condition influenced by various factors, including aging or specific physical conditions, with prolonged digital device use remaining the most significant risk factor [3,24].

Although some authors emphasize that DED or DES symptoms became more prevalent due to the new lifestyle adopted during the COVID-19 pandemic [3,13], a significant correlation between prolonged screen exposure and these symptoms had already been established in studies conducted prior to 2019, i.e., before the onset of the pandemic. Similarly, a study observing the general population did not find an increase in DES symptoms during the pandemic [28]. On the other hand, more recent studies highlight that excessive use of digital devices, as well as ocular surface disturbances, have persisted even after returning to a “normal routine” [17,29,30]. This contributes to the idea that the relationship between digital screen exposure and associated challenges in physical and mental health is not necessarily tied to the pandemic context but represents an integral part of life in modern society.

Discomfort and irritation of the ocular surface, as well as eye pain observed in both conditions, represent a significant stressor in a person’s life. Additionally, feelings of helplessness, frustration, and worry, which may be present in individuals with pronounced DED/DES symptoms, can lead to higher levels of depression and anxiety [22,31,32]. A significant association between ocular surface disturbances and mental health problems has been found in different population groups [21,22,27,33,34]. Studies conducted on eye clinic patients indicate a higher prevalence of depression and anxiety among individuals with pronounced DED symptoms [31,32]. This significant connection between DED or DES symptoms and depression and anxiety has also been observed in the general population [35]. Numerous studies have shown a significant association between the severity of DED/DES symptoms and the impact on daily activities, social functioning, and overall quality of life [8,34,35,36,37]. Individuals with more pronounced DED symptoms report greater difficulties with mobility, driving, work productivity, and the completion of daily tasks [32,36]. Furthermore, sleep quality is one of the key aspects of daily functioning that can be affected by the presence of DED or DES symptoms. Individuals with more pronounced symptoms are more prone to sleep disturbances, such as frequent awakenings during the night, and exhibit prolonged sleep latency, meaning it takes them longer to fall asleep [38]. Distress and depression caused by ocular surface disturbances may result in sleep difficulties, which can worsen DED symptoms and reciprocally worsen depression and anxiety [39,40].

The growing presence of digital technology in various aspects of our daily lives, further intensified by the changing circumstances during the COVID-19 pandemic, has led to prolonged exposure to digital devices among people of all ages. In addition to the significant impact on individuals’ work, social, and educational functioning, increased screen time is associated with decline in both physical health, especially eye health, and mental well-being.

While numerous studies have investigated the relationship between mental health, different aspects of sleep (such as quality and duration), and visual difficulties resulting from screen exposure, the direction of that interaction remains unclear. Therefore, the main objective of this paper is to provide a systematic review of existing literature to determine the complex interplay between digital activity, visual health, and subjective well-being. Additionally, focus will be on the relationship between digital screen use and ocular surface health, as well as the potential negative impact of impaired ocular surface health on levels of depression, anxiety, stress, and everyday functioning.

## 2. Materials and Methods

### 2.1. Study Selection

This systematic review was conducted following the PRISMA (Preferred Reporting Items for Systematic Review and Meta-Analysis Protocols) guidelines [41]. The electronic databases PubMed and Web of Science (WoS) were searched (from 16 December 2024 to 9 January 2025) to collect the publications relevant to the set aims. Only studies published within the last ten years (from 2014 to 2024) were considered to cover the period before and after the COVID-19 pandemic. Although the pandemic has increased researchers’ interest in investigating the association between exposure to digital devices and visual function, the importance of this topic was recognized earlier due to the development and growing presence of digital devices.

Selection of the studies for final analysis, including the evaluation of titles, abstracts, and ultimately the full text, as well as the quality assessment of the studies, was conducted independently by two authors of this paper (V.K. and D.K.). In the initial phase of the screening process, the titles and abstracts were assessed. In the second step, the full texts of the articles considered appropriate in the previous step were thoroughly examined. Additionally, the reference lists of the included articles were searched to detect other relevant publications. This review has been officially registered on Open OSF with the following https://doi.org/10.17605/OSF.IO/VPUCT.

### 2.2. Inclusion and Exclusion Criteria

To ensure the inclusion of studies that aligned closely with the objectives and scope of this systematic review, specific criteria were defined. This analysis included original scientific papers and is limited to those published in English and involving human participants. The following terms and their combinations were used in the database search: digital eye strain, computer vision syndrome, dry eye disease, screen use, depression, anxiety, and stress. Editorial letters, documents, dissertations, guidelines, preprints, and articles published only as abstracts were excluded from the analysis. In addition, we focused only on visual discomfort and ocular surface changes resulting from prolonged screen time. Therefore, papers that examined progressive ocular conditions characterized by physiological changes or damage to the eye, such as glaucoma or myopia, were excluded. Furthermore, studies that focused only on the development and validation of instruments for the assessment of digital eye strain were not included.

## 3. Results

**Figure 1:**
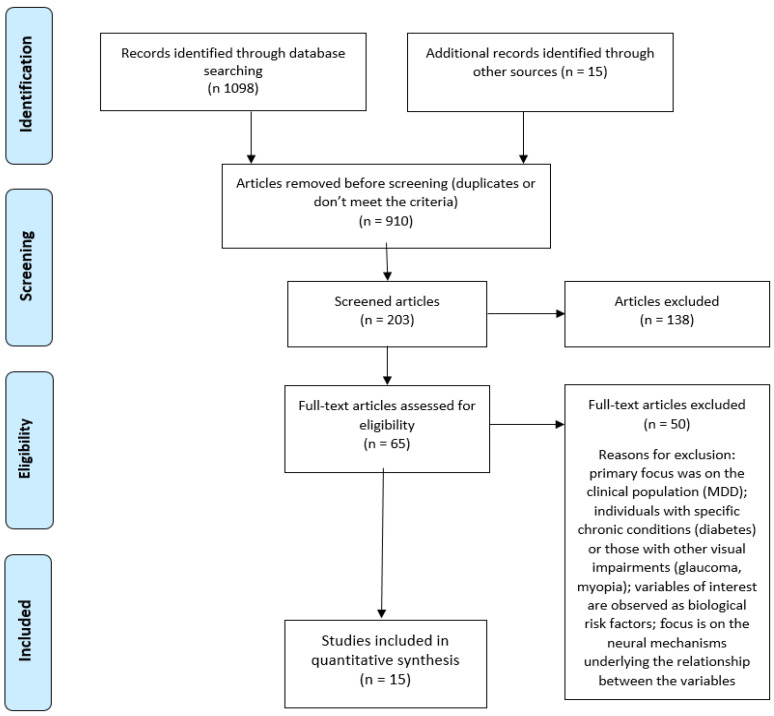
PRISMA flow diagram of included studies.

The process of screening and study selection is presented in the PRISMA diagram (Figure 1). The initial search of the PubMed and WoS databases yielded 1098 results, of which 910 were excluded due to repetition and non-compliance with the predefined criteria. Of the 203 papers whose titles and abstracts were analyzed in the first step of screening processes, only 65 were reviewed in their entirety. An additional 49 articles were excluded through full-text screening for reasons presented in Figure 1. The reference list of studies included in the analysis after the second screening was reviewed, with 15 additional relevant studies identified. After the full-text screening, only three additional studies were included in the final analysis. The process of screening and study selection led to the inclusion of 15 papers published in the online databases PubMed and WoS.

The characteristics of the studies included, along with their main findings, are presented in Table 1. All studies were cross-sectional and published between 2015 and 2024. Five studies collected data prior to 2019, i.e., before the COVID-19 pandemic [42,43,44,45,46], while the remainder were conducted and published during or after the pandemic. In total, 9 studies were conducted in Asia (56.3%), 5 in the Middle East (31.3%), 1 in Africa, and 1 in Europe (The Netherlands) (Table 1).

Considering the characteristics of the participants, five studies examined the relationship between the variables of interest in the general population, four in patients from eye clinics, five in the student population, and one focused on office workers who are regularly exposed to computer screens for extended periods of time (Table 1). Among the five studies examining the general population, two were population-based [44,45]. Out of the 15 studies included in the review, 3 involved participants under the age of 18 [42,47,54]. Two of these studies focused on high school and/or college students, while the third aimed to include individuals previously diagnosed with dry eyes or DED, regardless of age [42]. Most of the articles included in this study analyzed the severity of dry eye disease symptoms, whereas only four studies focused on symptoms of computer vision syndrome, i.e., digital eye strain (Table 1).

Depending on the population observed in the study, conclusions regarding the severity of DED symptoms or DES were based on different subjective and/or objective assessment measures. Publications that focused on patients in eye clinics included both self-assessment questionnaires and comprehensive ophthalmologic examinations (Table 1). An exception to this is the study by Ayaki et al. (2015) [43], which relied only on ophthalmologic examination to diagnose dry eye. Other publications categorized the participants and assessed the intensity and prevalence of the mentioned eye conditions based solely on self-assessment measures. In some cases, a validated questionnaire was used for assessment (e.g., [51,54,55]), with the Ocular Surface Disease Index (OSDI) being the most common, while in other cases, participants were only asked a few questions about the presence and frequency of symptoms such as irritation, dryness and redness (e.g., [44,45]). Studies that have used categorization based on the self-reported severity of DED or DES symptoms highlight that this approach is widely used and accepted, particularly in research involving the general population.

Cross-sectional studies examining the prevalence of eye- and vision-related issues have established a concerning presence of DED and DES symptoms, not only among eye-clinic patients [47,50,52] but also within the healthy population. According to research conducted on student populations, one-third of participants report experiencing, on average, at least six symptoms of ocular surface irritation [50], with the prevalence of severe symptoms among medical students reaching approximately 30% [55]. The most common ocular symptoms associated with prolonged digital device use were eye dryness, burning sensations, eye pain, and redness, while the most frequent non-ocular symptoms included headaches, neck stiffness, and lower back pain (e.g., [49,50]).

The severity and frequency of the reported eye and vision problems were analyzed in relation to the growing use of digital devices, whether for academic and professional activities or leisure, and entertainment. A cross-sectional study examining patients with DED [47] found that only a small proportion (12.6%) reported daily screen time of less than 5 hours, while 33.2% reported using their digital devices for 10 or more hours. An increase in dry eye symptoms associated with electronic device usage was observed in 86.8% of cases. Similar findings have been reported in studies conducted on the general population, where using digital devices for more than 8 h daily leads to a higher frequency and intensity of DED or DES symptoms [49,51]. Likewise, research on student populations found that longer durations of VDT use per day were associated with greater severity of digital eye strain (e.g., [50,55]).

The increase in digital screen use and the rising incidence of DED or DES observed across various populations have been associated with the circumstances of the COVID-19 pandemic, with most studies addressing this connection on a theoretical level [52,55]. An exception to this is a study conducted during the COVID-19 pandemic, which revealed that more than half of participants reported an increase in overall digital device usage by 5 h or more compared to the period before the pandemic, with around 40% being students. Additionally, over 50% of the participants reported that the frequency and intensity of these symptoms have increased since the lockdown measure was implemented [49].

Most of the included studies examined the relationship between ocular surface health and mental health, with differences noted in relation to participant characteristics (eye clinic patients, students, general population) and the observed psychological conditions. Out of 15 studies, 8 reported the relationship between the intensity of DED or DSD symptoms and depression, 7 examined their relationship with anxiety, and 7 studies reported on stress levels (Table 1). Findings from these studies indicate higher levels of depression, anxiety, and stress among individuals with greater frequency and intensity of DED or DSD symptoms. Research conducted prior to the COVID-19 pandemic indicates a significant positive association between DED or DES and symptoms of depression, anxiety, and stress (r > 0.04; *p* < 0.001; odds ratio, 1.5–2.7; e.g., [42,45]). A similar trend is observed in studies conducted during and after the pandemic, where higher frequency and severity of eye- and vision-related problems correlated with higher levels of depression, anxiety, and stress [53,54]. According to Alkozi et al. [48], objectively measured levels of stress (by cortisol levels in tears) were positively correlated with individuals’ experiences of dry eye symptoms (*p* < 0.05; r > 0.3). In contrast to the study by Sun et al. [56], whose findings indicate that the severity of DED does not have a significant influence on depression, the results of Asiedu et al. [42] suggest that the severity of DED has slightly stronger relationship with depressive symptoms (*p* < 0.001; r > 0.5), compared to other psychosomatic symptoms, such as anxiety and stress (*p* < 0.001; r = 0.4).

In addition to the previously mentioned indicators of mental health, studies have examined the relationship between the DED/DES symptoms and various aspects of quality of life. Poorer sleep quality, longer sleep latency, and greater difficulty falling asleep have been associated with the severity of DED symptoms. According to a study conducted on the general population [49], more than 60% of participants under the age of 26 experience sleep pattern disruptions due to increased digital device usage. Compared to diagnostic groups with other visual impairments, such as glaucoma, dry eye patients (*p* < 0.05) had the worst sleep duration, subjective quality of sleep, and highest daytime dysfunction [43]. Additionally, subjective sleep quality and sleep latency was found to be a mediator in the relationship of severity of DED with anxiety (*p* < 0.05) and depression (*p* < 0.01) [52]. A significant relationship was also identified between the frequency and intensity of DED or DES symptoms and impaired emotional and social functioning [44,54]. Individuals with more severe DED symptoms reported higher levels of ocular pain, greater concerns about eyesight, increased difficulties with driving, and more challenges in performing daily tasks [44,46].

## 4. Discussion

The findings from this review highlight the global and multifaceted impact of DED and DES on various populations, particularly in the context of increasing digital screen use. The included studies consistently report a high prevalence of DED and DES symptoms across diverse populations, with a notable increase during and after the COVID-19 pandemic. The majority of studies were conducted in Asia and the Middle East, reflecting regional differences in digital screen usage and environmental factors that may influence ocular surface health. Interestingly, while most studies focused on adults, three examined individuals under 18, emphasizing the early onset of these conditions, particularly among students exposed to prolonged screen time.

A key finding is the variability in assessment methods for DED and DES. While ophthalmologic examinations provide objective measures, most studies relied on self-reported symptoms, often using the Ocular Surface Disease Index (OSDI). This approach, while practical, may introduce subjectivity and limit the comparability of results [57]. Future research could benefit from integrating both subjective and objective measures to provide a comprehensive understanding of symptom severity and its impact.

The COVID-19 pandemic significantly accelerated digital screen use, with studies showing an increase in both frequency and severity of eye-related symptoms. These findings align with prior research linking prolonged digital device use to ocular discomfort [18]. Some reported that over half of participants experienced increased screen time by five or more hours daily during lockdowns, with a corresponding rise in DED and DES symptoms [49]. Most of the survey respondents declare using digital devices more than an average of 5 h daily. In addition, this finding is consistent with the 65% (n = 957) of participants reporting an increase in their use of digital devices from which 21.8% of them reported 4 h or more during the lockdown period [3]. In total, 88.1% of the participants were exposed to an average screen time of more than 4 h per day. An increased number of hours of digital device usage was found to be associated with higher total symptom scores. The total symptom scores were found to be considerably higher for those with continuous screen usage [2]. The physiological mechanisms underlying this relationship likely involve a decreased blink rate and increased ocular surface exposure during screen use, leading to tear film instability and evaporative dry eye [1]. These findings highlight the urgent need for preventive strategies, such as implementing regular screen breaks, promoting the 20-20-20 rule (every 20 min of work, look at least 20 s at a scene 20 feet away), and optimizing workplace ergonomics to mitigate the impact of prolonged screen exposure on ocular health [58]. This emphasizes the need for preventive strategies, such as promoting digital hygiene and ergonomic practices, particularly in educational and professional settings.

An important aspect of this review is the documented relationship between DED and DES symptoms and mental health outcomes, including depression, anxiety and stress. Studies consistently reported higher levels of these psychological symptoms in individuals with more severe ocular symptoms [22,31,32,33]. Significant difference in the prevalence and levels of physical and psychological health impact regarding the intensity of screen usage was found. A significant association between impaired mental health and individuals’ experiences of ocular dry eye symptoms or digital eye strain (computer vision syndrome), such as irritation, dryness, and blurriness, has been confirmed across various populations, ranging from eye clinic patients to students. Participants with a diagnosis of DED and DED symptoms were likely to have more depressive symptoms, anxiety problems and higher levels of pain/discomfort and psychological stress [40]. The bidirectional nature of this relationship warrants further investigation, as stress may exacerbate DED symptoms, creating a feedback loop.

Stress has been found to exacerbate DED symptoms through three key mechanisms: systemic inflammation, altered pain perception, and somatization. Psychological stress is known to upregulate the production of pro-inflammatory cytokines such as interleukin-6 (IL-6), tumor necrosis factor-alpha (TNF-α), and interleukin-1 beta (IL-1β) [59]. These cytokines contribute to a systemic inflammatory state, which plays a critical role in the pathogenesis of various chronic conditions, including DED. Inflammation of the ocular surface is a hallmark of DED, characterized by increased tear osmolarity, activation of epithelial stress pathways, and recruitment of immune cells [60,61]. Thus, the systemic inflammatory response induced by psychological stress may exacerbate the ocular surface inflammation seen in DED, leading to worsening symptoms such as dryness, burning, and ocular pain [22]. Psychological stress also affects pain perception, which may contribute to the heightened awareness of DED symptoms. Stress-induced activation of the HPA axis results in elevated cortisol levels, which have been shown to modulate pain sensitivity [62]. While cortisol has anti-inflammatory properties under normal conditions, chronic stress and prolonged cortisol exposure can lead to dysregulation of pain pathways. This dysregulation may increase central sensitization, making individuals more susceptible to experiencing ocular discomfort even in the absence of significant ocular surface damage [63]. A third mechanism by which psychological stress may exacerbate DED symptoms is through somatization, a process by which psychological distress manifests as physical symptoms. Somatization has been identified as a significant factor in chronic pain conditions, and its relevance to DED is increasingly recognized. Positive correlation was found between levels of perceived stress and somatization, suggesting that individuals with higher stress levels are more likely to experience and report physical symptoms, including ocular discomfort [64]. It is important to recognize the bidirectional relationship between ocular discomfort and psychological stress. Research indicates that even minor vision problems can negatively impact mental health, contributing to increased symptoms of psychological stress [65,66,67].

Apart from previously mentioned mental health outcomes, the severity of DED and DES symptoms has been associated with poorer sleep quality, sleep disturbances and impaired daily and social functioning. Prolonged sleep latency, short sleep duration, and sleep apnea were more commonly observed in patients with more frequent dry eye symptoms [38]. Previous studies also highlight a significant relationship between mood disorders, and impaired sleep quality in individuals with higher frequency and severity of DES/DED symptoms [43,68]. Distress, anxiety and depression caused by ocular surface disturbances may result in sleep difficulties, which can worsen DED symptoms and reciprocally worsen the mental health outcomes [39,40]. The impact of DED on quality of life, including diminished sleep quality and impaired social and emotional functioning, underscores the broader implications of these conditions.

### Limitations

This study has certain limitations that should be considered when interpreting the results. Only cross-sectional studies were included, which provide an incomplete picture of the relationship between ocular surface health and subjective well-being and limit the ability to draw conclusions about causal relationships. The observed publications also differ in terms of the populations studied, ranging from university students and general population to eye-clinic patients. Furthermore, the methodological inconsistencies in the studies should be emphasized, particularly with regard to the instruments used and the criteria for assessing the severity of DED or DES symptoms. These mentioned limitations restrict the ability to generalize the results and conclusions. Although this review focused primarily on examining the interaction between ocular surface disruptions due to prolonged screen exposure and mental health, there are other factors that may also be influenced by prolonged screen exposure and the associated physical symptoms. Future studies could further examine the effect of DED/DES symptom severity on daily activities, such as work productivity and social functioning, as well as the role of certain protective factors. Furthermore, the potentially bidirectional relationship between prolonged screen exposure and mental health was not observed in this study. Exploring the mentioned concepts could provide a deeper understanding of this increasingly important topic. In addition, future research should prioritize longitudinal studies across diverse populations to better understand causal relationships and explore potential interventions.

## 5. Conclusions

This review underscores the global burden and multifaceted impact of DED and DES, particularly in the context of rising digital screen use. The high prevalence of symptoms across diverse populations, intensified during the COVID-19 pandemic, highlights the urgent need for preventive strategies. Regional differences in digital habits and environmental factors were noted, as were the early onset of symptoms among youths exposed to prolonged screen time. Importantly, the established variability in assessment methods of the observed studies suggests that future research should combine subjective and objective measures for more robust findings. This review includes only cross-sectional studies. It would be valuable to examine the relationship between these constructs longitudinally and to explore the effectiveness of specific interventions. The strong association between DED and DES symptoms and mental health issues such as anxiety, depression, and stress further emphasize the systemic implications of these conditions. Addressing these challenges requires a holistic approach, integrating ergonomic interventions, digital hygiene, and mental health support to improve overall well-being and quality of life.

## Figures and Tables

**Table 1:** Characteristics of included studies.

Reference	Country	Study Design	Sample (% Male)	Mean Age (Years ± SD; Range)	Variables	Aim	Results	Conclusion
Al-Dairi et al., 2020 [47]	Saudi Arabia	Cross-sectional	General population with DED: 476 (male: 22.1)	22.7 ± 6.8	Dry eye symptoms; digital device usage; depression	Evaluate the relationship between DED and depression	An increase in dry eye symptoms associated with digital device usage was reported by 86.8% of the participants; depression was diagnosed among 42% of the participants with dry eyes, of which 36.4% had moderate or severe depression.	DED is associated with depression of higher degrees of severity. Impact of DED on the person’s daily activity is the reason behind the tendency of developing depression
Alkozi et al., 2024 [48]	Saudi Arabia	Cross-sectional	College students: 112 (male: 41.1%)	21.9 ± 1.7	Dry eye symptoms; anxiety; depression; stress	Investigate the relationships between ocular surface health and anxiety, depression and stress.	Experience of dry eye symptoms was significantly correlated with subjectively reported anxiety and depression, as well as the stress levels.	Link between anxiety and depression in young people in good health and dry eye symptoms was established. Investigating the relationship between stress and various health diseases, such as dry eye disease, is of immense value for gaining new insights.
Asiedu et al., 2018 [42]	Africa	Cross-sectional	Eye clinic patients: 211 (male: 49.7%)	21.7 ± 3.0	DED severity; depression; anxiety; stress; QoL	Determine the impact of dry eye on quality of life, depression, anxiety, and stress.	Statistically significant association of dry eye symptoms with stress, anxiety, and depression was found. Anxiety, stress and depression increased significantly when dry eye symptoms became more severe. Dry eye symptoms, stress, anxiety, and depression correlated with quality life scores.	Severity of dry eye symptoms impacted on psychosomatic symptoms and quality of life. Psychosomatic symptoms may play a role in the poor quality of life of youth with dry eye symptoms. Clinicians should consider addressing symptomatic dry eye more holistically.
Ayaki et al., 2015 [43]	Japan	Cross-sectional	Eye clinic patients: 730 (male: 33.3%) Group with DED: 247 (male: 17.8%)	59.5 61.4	Quality of sleep/sleep disorders; anxiety; depression	Investigate the prevalence of sleep and mood disorders in six diagnostic groups with different ocular problems.	Patients with dry eye had the lowest quality of sleep, i.e., the worst scores on sleep duration, subjective sleep, and daytime dysfunction; HADS scores for anxiety and depression were highest or second-highest in the dry eye group.	Prevalence of sleep and mood disorders was highest in patients with dry eye, with some of these patients showing clinically significant scores for sleep disturbances and anxiety and depression.
Bahkir & Grandee, 2020 [49]	India	Cross-sectional	General population: 407 (male: 55.5%)	27.4 58% (19–26 years)	Digital device usage; ocular surface health; sleep	Evaluate the relationship between digital device usage after COVID-19 and its effect on ocular surface health and circadian rhythm.	95.8% of participants experienced at least one symptom related to digital device usage; sleep pattern disruption due to increased digital device usage was found in more than 60% of participants; increase in screen time was related to more intense symptoms of digital eye strain and more frequent sleep disturbances.	Increase in digital device usage after the COVID-19 lockdown led to deterioration of ocular health across all age groups, as well as sleep disturbances.
Bin Maneea et al., 2024 [50]	Saudi Arabia	Cross-sectional	College students: 203 (male: 18.2)	18–25 age range: 76.6%	Digital device usage; symptoms of DES; quality of life	Assess prevalence, awareness, risk factors of DES, and its effect on quality of life.	The majority (41.6%) reported using digital devices for six to eight hours per day. Almost one third reported 6 to 10 symptoms of DES. Significant association was found between using digital devices and symptom severity; symptom severity affected productivity, attention and the ability to complete tasks.	There is a significant relationship between the incidence of digital eye straining and longer screen exposure time. The results contribute to the broader discourse on digital eye health and emphasize the need for targeted interventions to alleviate the impact of DES on daily life.
Das et al., 2022 [51]	Nepal	Cross-sectional	Office workers: 319 (male: 71.6%)	33.4 ± 9.4 years	CVS; musculoskeletal problems; work-related stress	Estimate the prevalence of CVS-DES, musculoskeletal and work-related stress among VDT screen users.	More than eight out of ten participants reported at least one visual and musculoskeletal symptom; using the VDT screen for more than 8 h a day was associated with presence of CVS symptoms, as well as higher level of stress and musculoskeletal problems.	With the increase in time spent in front of the computer, the presence of visual symptoms of CVS, musculoskeletal symptoms, and work-related stress also increases.
He et al., 2022 [52]	China	Cross-sectional	Patients with DED: 321 (male: 27.7%)	48.41	Severity of self-reported DED; anxiety; depression; sleep quality	Investigate the relationship between DED and anxiety and depression, as well as the mediating role of sleep quality in this relationship.	More severe DED (i.e., higher OSDI scores) was associated with poorer sleep quality, longer sleep latency, and depressive and anxiety symptoms; relationship between DED severity and anxiety and depression was mediated by sleep quality and sleep latency.	Many patients with DED experience anxiety, depression, and sleep disorders due to eye discomfort symptoms.
Issa et al., 2023 [53]	Lebanon	Cross-sectional	General population: 749 (male: 34.4%)	24.5 ± 7.7	CVS; mental health (depression, anxiety, stress)	Determine the association between CVS and depression and anxiety among a sample of Lebanese young adults, along with evaluating the mediating effect of stress on these associations.	Higher frequency and severity of CVS symptoms was associated with higher levels of depression, anxiety and stress.	Findings could serve as a starting point for healthcare providers and psychologists to look deeper into CVS when looking for reasons behind mental health issues.
Morthen et al., 2022 [44]	Netherlands	Cross-sectional	General population: 89.022 (male: 41%)	50.7 ± 13.0	DED; vision-related quality of life (VR-QoL)	Investigate the relationship between DED and vision-related quality of life (VR-QoL) at population level.	Frequency and intensity of the DED symptoms was associated with a substantial reduction in all domains of VR-QoL, i.e., with reduction in near vision, distance vision, social functioning, and driving, and with increased ocular pain and worrying about eyesight.	Dry eye was associated with a substantial reduction in all domains of VR-QoL investigated. It should be emphasized to clinicians and the general public that dry eye is a serious disorder that must be adequately diagnosed and treated.
Na et al., 2015 [45]	Korea	Cross-sectional	General population: 6655 women	19 years and older (mean range 44.9–46.5)	DED; DED symptoms; depression; anxiety; stress	Determine the relationship between DED and depressive symptoms, psychological stress and anxiety problems.	Participants with a diagnosis of DED and DED symptoms were likely to have more depressive symptoms, anxiety problems and higher levels of pain/discomfort and psychological stress.	There is a close association between depression, stress, and DED in women who have been clinically diagnosed with it or those presenting with its symptoms. Ophthalmologists must familiarize themselves with the prevalence of psychological problems in patients with individuals with clinically diagnosed or symptomatic DED
Rao et al., 2024 [54]	India	Cross-sectional	Students from high schools and colleges: 120 (male: 66.6%)	19.3 ± 3.2 years	Screen use; mental health (depression, anxiety, stress, loneliness); physical health (DED; sleep quality, back/neck pain)	Determine the prevalence and severity of physical and psychological health impact in screen users with addictive behaviour compared to healthy screen users, and to explore association between these health effects and screen usage.	Unhealthy screen users had greater severity of DED and more musculoskeletal and sleep-related problems, due to prolonged exposure to screen devices; use of digital devices for extended periods of time with higher levels of depression, anxiety, stress, loneliness and aggressive behaviour.	These results could increase awareness on the health effects caused by prolonged use of screen devices. Associations between a variety of health effects and prolonged screen usage suggest that not only should standard diagnostic criteria be formulated for diagnosing screen addiction, but these health implications should also be included as a part of the diagnostic tool.
Tangmonkongvoragul et al., 2022 [55]	Thailand	Cross-sectional	College students: 528 (male: 47.4%)	20.48 (17–31) years	Dry eye symptoms; psychological stress; contact lens use; duration of VDT use	Explore the prevalence and potential risk factors of DED among medical students.	High prevalence of symptomatic DED in medical students (70.8%); psychological stress, contact lens use, and prolonged duration of VDT use had a significant correlation with increased severity and risk of DED.	Most of the risk factors (stress, contact lens, VDT use) were modifiable and may be used as initial management in patients with DED.
Tananuvat et al., 2022 [46]	Thailand	Cross-sectional	Eye-clinic patients: 100 (male: 11%)	50.9 ± 14.4 years	DED symptoms; quality of life (DED specific and general health-related QOL), stress; neuroticism.	Examine how psychological factors, i.e., perceived stress, and the personality trait of neuroticism impacted the relationship between dry eye symptoms and QOL	Intensity of the DED symptoms had significant impact on daily life as well as general health-related QOL (mobility, self-care, usual activities, pain/discomfort and anxiety/depression); QOL of the patients with DED, is not only related to eye symptoms but also perceived stress.	Incorporating screening for perceived stress and neuroticism into routine practice can assist clinicians in developing more effective treatment plans aimed at enhancing the quality of life for individuals experiencing DED symptoms.
Sun et al., 2024 [56]	Philippines	Cross-sectional	Doctoral students: 120 (male: 31%)	36.3 ± 7.0	DED severity; depression; anxiety; stress	This study aimed to evaluate the impact of DED severity on depression, anxiety, and stress	Levels of depression, anxiety and stress were significantly higher in students that exhibited more DED symptoms. DED severity had a significant impact on anxiety and psychological stress but did not show a significant influence on depression	Anxiety and stress levels increase with the severity of DED.

## Data Availability

Data are contained within the article.
